# Morphological and phylogenetic characterisations reveal three new species of *Samsoniella* (Cordycipitaceae, Hypocreales) from Guizhou, China

**DOI:** 10.3897/mycokeys.74.56655

**Published:** 2020-10-19

**Authors:** Wan-Hao Chen, Yan-Feng Han, Jian-Dong Liang, Wei-Yi Tian, Zong-Qi Liang

**Affiliations:** 1 Basic Medical School, Guizhou University of Traditional Chinese Medicine, Guiyang 550025, Guizhou, China Guizhou University of Traditional Chinese Medicine Guiyang China; 2 Institute of Fungus Resources, Department of Ecology, College of Life Sciences, Guizhou University, Guiyang 550025, Guizhou, China Guizhou University Guiyang China

**Keywords:** Isaria-like, morphology, nutritional preference, phylogeny

## Abstract

*Samsoniella* species have been found on lepidopteran larvae or pupae buried in soil or leaf litter. Three new species, *Samsoniella hymenopterorum*, *S. coleopterorum* and *S. lepidopterorum*, parasitic on hymenopteran larvae, coleopteran larvae and lepidopteran pupae, respectively, are reported. Morphological comparisons with extant species and DNA-based phylogenies from analysis of a multigene (ITS, *RPB1*, *RPB2* and *TEF*) dataset supported the establishment of the new species. Unusually, all three new species have mononematous conidiophores. The new species are clearly distinct from other species in *Samsoniella* occurring in separate subclades.

## Introduction

The genus *Isaria* Pers. was introduced for entomogenous fungi with mononematous or synnematous conidiophores, usually consisting of several verticillate branches, each bearing a dense whorl of phialides characters. The phialides consist of a cylindrical or swollen basal portion, terminating in a thin, often long neck and produce divergent conidial chains (Samson 1974). However, entomogenous species, morphologically similar to *Isaria*, can be found distributed throughout the Hypocreales (Luangsa-ard et al. 2004).

Kepler et al. (2017) proposed the rejection of *Isaria* in favour of *Cordyceps*, owing to the confusion surrounding the application of *Isaria* and combined 11 species into *Cordyceps*. Mongkolsamrit et al. (2018) described some Isaria-like species and proposed the new genus *Samsoniella* Mongkols., Noisrip., Thanakitp., Spatafora & Luangsa-ard. The typical characteristics of *Samsoniella* are oval to fusiform conidia and bright red-orange teleomorphic stromata and anamorphic synnemata. *Samsoniella* species inhabit lepidopteran larvae and pupae in leaf litter or soil. Currently, *Samsoniella* consists of three species, *S. alboaurantia* (G. Sm.) Mongkols., Noisrip., Thanakitp., Spatafora & Luangsa-ard, *S. aurantia* Mongkols., Noisrip., Thanakitp., Spatafora & Luangsa-ard and *S. inthanonensis* Mongkols., Noisrip., Thanakitp., Spatafora & Luangsa-ard.

Three infected insect specimens were collected during a survey of entomogenous fungi in south-western China. Morphological and molecular phylogenetic analyses suggested that these isolates represented three new species, which are described here as *Samsoniella hymenopterorum* sp. nov., *S. coleopterorum* sp. nov. and *S. lepidopterorum* sp. nov.

## Materials and methods

### Specimen collection and identification

Three fungus-infected insect specimens were collected from Xishui County (28°29'56.70"N, 106°24'31.04"E) (A1950 and A1952) and Dali, Rongjiang County (26°01'58.70"N, 108°24'48.06"E) (DL1007), Guizhou Province, on 20 July and 1 October 2018, respectively. Isolation of the fungi was done as described by Chen et al. (2019). The surface of the specimens was rinsed with sterile water, followed by surface sterilisation with 75% ethanol for 3–5 sec. A part of the insect body was cut off and inoculated with haemocoel on potato dextrose agar (PDA) and PDA, to which 1% w/v peptone (PDAP) had been added. Fungal colonies emerging from specimens were isolated and cultured at 22 °C for 14 d under 12 h light/12 h dark conditions following protocols described by Zou et al. (2010). Accordingly, strains A19501, A19502, A19521, A19522, DL10071 and DL10072 were obtained. The specimens and the isolated strains were deposited in the Institute of Fungus Resources, Guizhou University (formally Herbarium of Guizhou Agricultural College; code, GZAC), Guiyang City, Guizhou, China.

Macroscopic and microscopic morphological characteristics of the fungi were examined and growth rates determined from PDA cultures incubated at 25 °C for 14 d. Hyphae and conidiogenous structures were mounted in lactophenol cotton blue or 20% lactate solution and observed with an optical microscope (OM, DM4 B, Leica, Germany).

### DNA extraction, PCR amplification and nucleotide sequencing

DNA extraction was carried out in accordance with Liang et al. (2011). The extracted DNA was stored at −20 °C. Translation elongation factor 1 alpha (*TEF*) and RNA polymerase II largest subunit 2 (*RPB2*) genes were amplified using 983F/2218R and RPB2-5F/RPB2-7Cr primers, according to van den Brink et al. (2012). The RNA polymerase II largest subunit 1 (*RPB1*) gene was amplified with the primer pair CRPB1 and RPB1-Cr (Castlebury et al. 2004). The internal transcribed spacer (ITS) region was amplified by PCR using ITS4/ITS5, which was described by White et al. (1990). PCR products were purified using the UNIQ-10 column PCR products purification kit (no. SK1141; Sangon Biotech (Shanghai) Co., Shanghai, China) in accordance with the manufacturer’s protocol and sequenced at Sangon Biotech (Shanghai) Co. The resulting sequences were submitted to GenBank.

### Sequence alignment and phylogenetic analyses

The DNA sequences, generated in this study, were assembled and edited using Lasergene software (version 6.0 DNASTAR). Generated ITS, *RPB1*, *RPB2* and *TEF* sequences were aligned with those published by Mongkolsamrit et al. (2018) and others selected on the basis of BLAST algorithm-based searches in GenBank (Table 1). *Purpureocillium lilacinum* (Thom) Luangsa-ard, Houbraken, Hywel-Jones & Samson (isolates CBS 284.36 and CBS 431.87) and *Beauveria bassiana* (Bals.-Criv.) Vuill. (ARSEF 1564) were chosen as outgroup taxa for the analysis of *Samsoniella* in Cordycipitaceae and *Samsoniella* species and closely-related species, respectively. Multiple datasets of ITS, *RPB1*, *RPB2* and *TEF* were aligned using MAFFT v7.037b (Katoh and Standley 2013) and alignments were edited with MEGA6 (Tamura et al. 2013). Sequences were concatenated with SequenceMatrix v.1.7.8 (Vaidya et al. 2011). The partition homogeneity test in PAUP4.0b10 (Swofford 2002) was undertaken by using the command ‘hompart’.

**Table 1.** Taxa included in the phylogenetic analyses.

Species	Strain No.	GenBank Accession No.			
		ITS	RPB1	RPB2	TEF
*Akanthomyces aculeatus*	HUA 772	KC519371			KC519366
*А. attenuatus*	CBS 402.78	AJ292434	EF468888	EF468935	EF468782
*А. coccidioperitheciatus*	NHJ 6709	JN049865	EU369067	EU369086	
*А. farinosa*	CBS 541.81	AY624180			JQ425686
*А. kanyawimiae*	TBRC 7242	MF140751	MF140784	MF140808	MF140838
	TBRC 7243	MF140750	MF140783	MF140807	MF140837
	TBRC 7244	MF140752			MF140836
*А. lecanii*	CBS 101247	JN049836	DQ522407	DQ522466	DQ522359
*А. sulphureus*	TBRC 7247	MF140756	MF140785	MF140811	MF140841
	TBRC 7248	MF140758	MF140787	MF140812	MF140843
	TBRC 7249	MF140757	MF140786	MF140734	MF140842
*А. thailandicus*	TBRC 7245	MF140754		MF140809	MF140839
	TBRC 7246	MF140755		MF140810	MF140840
*А. tuberculatus*	BCC 16819	GQ250012			GQ250037
	OSC111002	JN049830	DQ522384	DQ522435	DQ522338
*А. waltergamsii*	TBRC 7250	MF140749			MF140835
	TBRC 7251	MF140747	MF140781	MF140805	MF140833
	TBRC 7252	MF140748	MF140782	MF140806	
*Ascopolyporus polychrous*	P.C. 546		DQ127236		DQ118745
*Beauveria acridophila*	HUA 179219		JX003857	JX003841	JQ958613
*B. acridophila*	QCNE 186726	JQ958605	JX003855		JQ958618
*B. bassiana*	ARSEF 1564	HQ880761	HQ880833	HQ880905	HQ880974
*B. brongniartii*	ARSEF 617	HQ880782	HQ880854	HQ880926	HQ880991
	BCC 16585	JN049867	JN049885	JF415991	JF416009
*B. caledonica*	ARSEF 2567	HQ880817	EF469086	HQ880961	EF469057
*B. diapheromeriphila*	MCA 1557	JQ958608	JX003851		JQ958612
	QCNE 186272	JQ958599	JX003848		JQ958610
	QCNE 186714	JQ958603	JX003850		JQ958611
*B. locustiphila*	HUA 179217	JQ958609	JX003847		
	HUA 179218	JQ958606	JX003846	JX003845	JQ958619
*B. malawiensis*	ARSEF 7760		HQ880897	HQ880969	DQ376246
*B. pseudobassiana*	ARSEF 3405	AY532022	HQ880864	HQ880936	AY531931
*B. scarabaeidicola*	ARSEF 5689	JN049827	DQ522380	DQ522431	DQ522335
*B. staphylinidicola*	ARSEF 5718		EF468881		EF468776
*Blackwellomyces cardinalis*	OSC 93609		DQ522370	DQ522422	DQ522325
*B. cardinalis*	OSC 93610	JN049843	EF469088	EF469106	EF469059
*B. pseudomilitaris*	NBRC 101409	JN943305	JN992482		
	NBRC 101410	JN943307	JN992481		
*Cordyceps amoene-rosea*	CBS 729.73			MG665235	HM161732
*C. amoene-rosea*	CBS 107.73	AY624168			
*C. bifusispora*	EFCC 5690		EF468854	EF468909	
	EFCC 8260		EF468855	EF468910	EF468747
*C. blackwelliae*	TBRC 7253	MF140739	MF140774	MF140798	MF140825
	TBRC 7254	MF140738	MF140773	MF140797	MF140824
	TBRC 7255	MF140737	MF140772	MF140796	MF140823
	TBRC 7256	MF140736	MF140771	MF140795	
	TBRC 7257	MF140735	MF140770	MF140794	MF140821
*C. cateniannulatus*	CBS 152.83	AY624172			JQ425687
	TBRC 7258	MF140753	MF140767		MF140850
*C. cateniobliqua*	CBS 153.83	AY624173		MG665236	JQ425688
C. cf. farinosa	OSC 111004		EF468886		EF468780
C. cf. ochraceostromata	ARSEF 5691		EF468867	EF468921	EF468759
C. cf. takaomontana	NHJ 12623		EF468884	EF468932	EF468778
*C. chiangdaoensis*	TBRC 7274	KT261393			KT261403
*C. coleopterorum*	CBS 110.73	AY624177	JN049903	JF416006	JF416028
*C. farinosa*	CBS 111113	AY624181		GU979973	GQ250022
*C. fumosorosea*	CBS 107.10	AY624184		MG665237	HM161735
	CBS 244.31	AY624182			JQ425690
	CBS 375.70	AY624183		MG665238	HM161736
	CBS 337.52	EF411219			MG665233
*C. javanica*	CBS 134.22	AY624186			JQ425683
	TBRC 7259	MF140745	MF140780	MF140804	MF140831
	TBRC 7260	MF140744	MF140779	MF140803	MF140830
	TBRC 7261	MF140743	MF140778	MF140802	MF140829
	TBRC 7262	MF140746			MF140832
*C. kintrischica*	ARSEF 7218	EU553278			GU734751
	ARSEF 8058	GU734764			GU734750
*C. kyusyuensis*	EFCC 5886		EF468863	EF468917	
*C. lepidopterorum*	TBRC 7263	MF140765	MF140768	MF140792	MF140819
	TBRC 7264	MF140766	MF140769	MF140793	MF140820
*C. militaris*	OSC 93623				
*C. morakotii*	TBRC 7275	KT261388			KT261398
	TBRC 7276	KT261390			KT261400
*C. ninchukispora*	EFCC 5197		EF468868		EF468760
	EFCC 5693		EF468869		EF468762
	EGS 38.165		EF468900		EF468795
	EGS 38.166		EF468901		EF468794
	NHJ 10627		EF468870		EF468763
	NHJ 10684		EF468871		EF468761
*C. oncoperae*	ARSEF 4358		EF468891	EF468936	EF468785
*C. piperis*	CBS 116719		DQ127240	EU369083	DQ118749
*C. pruinosa*	ARSEF 5413	JN049826	DQ522397	DQ522451	DQ522351
*Cordyceps* sp.	CBS 102184		EF468907	EF468948	EF468803
*C. takaomontana*	BCC 28612	FJ765285			FJ765268
*C. tenuipes*	ARSEF 5135	AY624196	JN049896	JF416000	JF416020
	OSC 111007		DQ522395	DQ522449	DQ522349
	TBRC 7265	MF140741	MF140776		MF140827
	TBRC 7266	MF140742	MF140777	MF140801	MF140828
	TBRC 7267	MF140740	MF140775	MF140799	MF140826
*Engyodontium aranearum*	CBS 309.85		DQ522387	DQ522439	DQ522341
*Gibellula longispora*	NHJ 12014		EU369055	EU369075	EU369017
*G. ratticaudata*	ARSEF 1915		DQ522408	DQ522467	DQ522360
*Gibellula* sp.	NHJ 10788		EU369058	EU369078	EU369019
	NHJ 13158		EU369057	EU369077	EU369020
	NHJ 10808		EU369056	EU369076	EU369018
	NHJ 5401		EU369059	EU369079	
	NHJ 7859		EU369064	EU369085	
*Hevansia cinerea*	NHJ 3510		EU369048	EU369070	EU369009
*H. nelumboides*	BCC 41864	JN201871			JN201867
*H. novoguineensis*	NHJ 4314		EU369051	EU369071	EU369012
	NHJ 10469		EU369047		EU369008
	NHJ 11923		EU369052	EU369072	EU369013
	NHJ 13117		EU369049	EU369073	EU369010
	NHJ 13161		EU369050		EU369011
*Hyperdermium pulvinatum*	P.C. 602		DQ127237		DQ118746
*Lecanicillium aranearum*	CBS 350.85		DQ522396	DQ522450	DQ522350
*L. aranearum*	CBS 726.73a		EF468887	EF468934	EF468781
*L. fusisporum*	CBS 164.70		EF468889		EF468783
*L. psalliotae*	CBS 101270		EF469095	EF469113	EF469066
	CBS 363.86		EF468890		EF468784
	CBS 532.81		EF469096	EF469112	EF469067
*Purpureocillium lilacinum*	CBS 284.36	AY624189	EF468792	EF468898	EF468941
*P. lilacinum*	CBS 431.87	AY624188	EF468897	EF468940	EF468791
*Samsoniella alboaurantium*	CBS 240.32	AY624178	JN049895	JF415999	JF416019
*S. alboaurantium*	CBS 262.58	AY624179			JQ425685
*S. aurantia*	TBRC 7271	MF140764	MF140791	MF140818	MF140846
	TBRC 7272	MF140763		MF140817	MF140845
	TBRC 7273	MF140762		MF140816	MF140844
***S. coleopterorum***	**A19501**	**MT626376**	**MT642600**	**MN101585**	**MN101586**
	**A19502**	**MT626625**	**MT642603**	**MN101587**	**MT642602**
***S. hymenopterorum***	**A19521**	**MN128224**	**MT642601**	**MT642604**	**MN101588**
	**A19522**	**MN128081**	**MN101589**	**MN101590**	**MN101591**
*S. inthanonensis*	TBRC 7915	MF140761	MF140790	MF140815	MF140849
	TBRC 7916	MF140760	MF140789	MF140814	MF140848
	TBRC 7270	MF140759	MF140788	MF140813	MF140847
***S. lepidopterorum***	**DL10071**	**MN128076**	**MN101592**	**MN101593**	**MN101594**
	**DL10072**	**MN128084**		**MT642605**	**MT642606**
*Simplicillium lamellicola*	CBS 116.25	AJ292393	DQ522404	DQ522462	DQ522356
*S. lanosoniveum*	CBS 101267	AJ292395	DQ522405	DQ522463	DQ522357
	CBS 704.86		DQ522406	DQ522464	DQ522358
*Torrubiella wallacei*	CBS 101237		EF469102	EF469119	EF469073

Maximum Likelihood (ML) analyses were constructed with RAxMLGUI (Silvestro and Michalak 2012). The GTRGAMMA model was used for all partitions, in accordance with recommendations in the RAxML manual against the use of invariant sites. For Bayesian Inference (BI), a Markov Chain Monte Carlo (MCMC) algorithm was used to generate phylogenetic trees with Bayesian probabilities using MrBayes v.3.2 (Ronquist et al. 2012) for the combined sequence datasets. The selection of the best-fit nucleotide substitution model for each locus was calculated by the Akaike Information Criterion (AIC) with jModelTest 2 (Darriba et al. 2012). The TIM+I+G model was selected for the concatenated ITS+*RPB1*+*RPB2*+*TEF* sequences. The Bayesian analysis resulted in 20,001 trees after 10,000,000 generations. The first 4,000 trees, representing the burn-in phase of the analyses, were discarded, while the remaining 16,001 trees were used for calculating posterior probabilities in the majority rule consensus tree. After the analysis was finished, each run was examined using the programme Tracer v1.5 (Drummond and Rambaut 2007) to determine burn-in and confirm that both runs had converged. The final alignment is available from TreeBASE under submission ID: 24710 (http://www.treebase.org).

## Results

### Phylogenetic analyses

**Figure 1.**
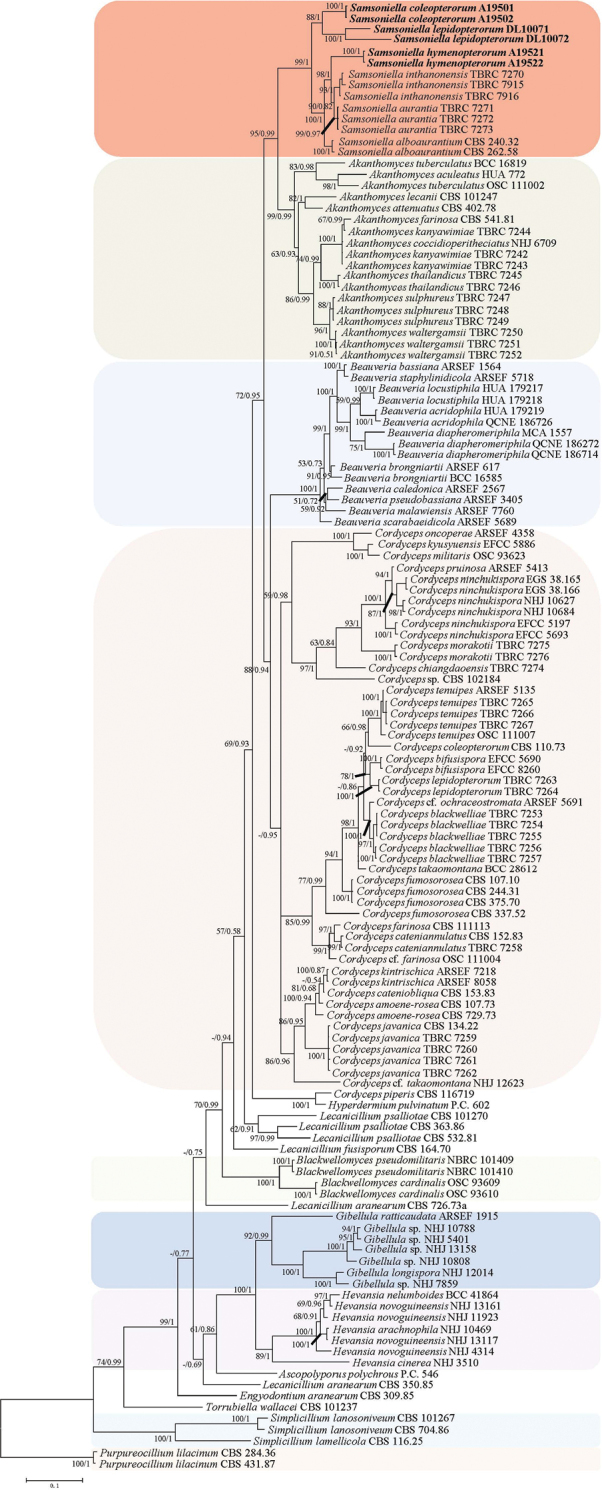
Phylogenetic relationships of the genus *Samsoniella* in Cordycipitaceae, based on multigene dataset (ITS, *RPB1*, *RPB2* and *TEF*). Statistical support values (≥ 50%/0.5) are shown at the nodes for ML bootstrap support/BI posterior probabilities.

**Figure 2.**
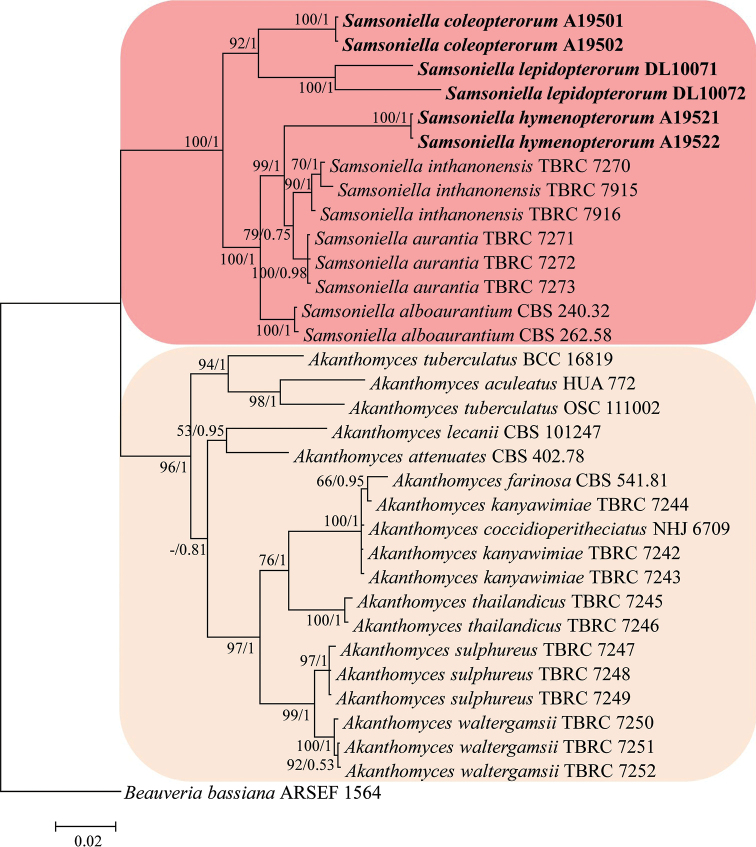
Phylogenetic relationships between the genus *Samsoniella* and closely-related species, based on multigene dataset (ITS, *RPB1*, *RPB2* and *TEF*). Statistical support values (≥ 50%/0.5) are shown at the nodes for ML bootstrap support/BI posterior probabilities.

The phylogenetic tree of *Samsoniella* in Cordycipitaceae (Fig. 1) and *Samsoniella* species and closely related species (Fig. 2) were generated from the ML and BI analysis, based on a combined data set of ITS, *RPB1*, *RPB2* and *TEF* sequence data. Statistical support (≥ 50%/0.5) is shown at the nodes for ML bootstrap support/BI posterior probabilities (Figs 1, 2). The strain numbers are noted after each species’ name. The concatenated sequences of analysis 1 and analysis 2 included 67 and 17 taxa, and consisted of 2,152 (ITS: 528, *RPB1*: 488, *RPB2*: 442 and *TEF*: 694) and 2,194 (ITS: 477, *RPB1*: 565, *RPB2*: 473 and *TEF*: 679) characters with gaps, respectively.

Analysis 1: *Samsoniella* in Cordycipitaceae. The RAxML analysis of the combined dataset (ITS+*RPB1*+*RPB2*+*TEF*) yielded a best scoring tree (Fig. 1) with a final ML optimisation likelihood value of –28,809.222105. Parameters for the GTR model of the concatenated dataset was as follows: estimated base frequencies; A = 0.234094, C = 0.301291, G = 0.260521, T = 0.204093; substitution rates AC = 1.111784, AG = 3.130020, AT = 0.930972, CG = 0.886915, CT = 6.300092, GT = 1.000000; gamma distribution shape parameter α = 0.390179. In the phylogenetic tree (Fig. 1), *Samsoniella* species were clustered in a clade and resolved into two obvious clades. *Samsoniella* species have a close relationship with *Akanthomyces* species.

Analysis 2: *Samsoniella* species and closely-related species. The RAxML analysis of the combined dataset (ITS+*RPB1*+*RPB2*+*TEF*) yielded a best scoring tree (Fig. 2) with a final ML optimisation likelihood value of –9,722.503130. Parameters for the GTR model of the concatenated data set were as follows: estimated base frequencies; A = 0.233473, C = 0.298686, G = 0.261629, T = 0.206212; substitution rates AC = 1.250081, AG = 2.534760, AT = 0.891128, CG = 0.827805, CT = 5.916085, GT = 1.000000; gamma distribution shape parameter α = 0.674468. In the phylogenetic tree (Fig. 2), *Samsoniella* species were clustered in a clade and easily distinguished with *Akanthomyces* species. *S. coleopterorum* and *S. lepidopterorum* clustered in a clade (Fig. 2) and formed two independent branches. *S. hymenopterorum* was phylogenetically close to *S. inthanonensis* and *S. aurantia*.

## Taxonomy

### Samsoniella coleopterorum

Taxon classificationFungiHypocrealesCordycipitaceae

W.H. Chen, Y.F. Han & Z.Q. Liang sp. nov.

629653D9-9471-5715-9DB0-8FBB3FDE9DD7

831735

Fig. 3

#### Diagnosis.

Differs from *Samsoniella aurantia* by having smaller conidia and snout beetle host in the family Curculionidae. Differs from *S. lepidopterorum* by having cylindrical to ellipsoidal phialides, smaller fusiform to ellipsoidal conidia and a different host.

**Figure 3.**
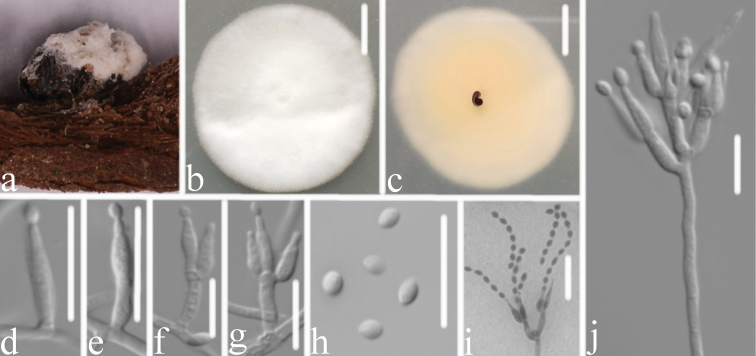
*Samsoniella coleopterorum***A** infected insect (Coleoptera) **B, C** top (**B**) and underside (**C**) of a colony cultured on PDA medium at 14 d **D, E, F, G, I** phialides and conidia in chains **J** conidiophore and phialides **H** conidia. Scale bars: 10 mm (**B, C**); 10 μm (**D–J**).

#### Type.

China, Guizhou Province, Xishui County (28°29'56.70"N, 106°24'31.04"E), July 2018, Jiandong Liang, holotype GZAC A1950, ex-type culture GZAC A19501. Sequences from isolated strain A19501 have been deposited in GenBank with accession numbers: ITS = MT626376, *RPB1* = MT642600, *RPB2* = MN101585 and *TEF* = MN101586.

#### Description.

Colonies on PDA, 3.6–4.0 cm diam. in 14 d at 25 °C, white, consisting of a basal felt and cottony, floccose hyphal overgrowth, reverse yellowish. Prostrate hyphae smooth, septate, hyaline, 1.1–1.8 μm diam. Erect conidiophores usually arising from aerial hyphae, Isaria-like with phialides in whorls of two to four. Phialides 5.4–9.7 × 1.2–1.8 μm, with a cylindrical to ellipsoidal basal portion, tapering into a short distinct neck. Conidia in chains, hyaline, fusiform, ellipsoidal or subglobose, one-celled, 1.7–2.5 × 1.2–1.8 μm. Chlamydospores and synnemata not observed. Size and shape of phialides and conidia similar in culture and on natural substratum. Sexual state not observed.

#### Host.

Snout beetle, family Curculionidae.

#### Distribution.

Xishui County, Guizhou Province, China.

#### Etymology.

Referring to its insect host, order Coleoptera.

#### Remarks.

*Samsoniella coleopterorum* was easily identified as belonging to *Samsoniella* based on the phylogenetic analyses (Fig. 1). Comparing with the typical characteristics of three species (Table 2), *S. coleopterorum* has a close relationship with *S. aurantia* by having cylindrical to ellipsoidal phialides and similar in size. However, it differs from *S. aurantia* by having shorter conidia and snout beetle host in the family Curculionidae. Based on the combined dataset of ITS, *RPB1*, *RPB2* and *TEF* sequences, *S. coleopterorum* has a close relationship with *S. lepidopterorum* (Fig. 2). However, *S. coleopterorum* has cylindrical to ellipsoidal phialides, smaller fusiform to ellipsoidal conidia and a different host.

**Table 2.** Morphological comparison of three new species with other *Samsoniella* species.

Species	Morphological characteristics			Reference
	Phialide (μm)	Conidia (μm)	Hosts/substrates	
*Samsoniella alboaurantium*	5–8 × 2	ovate to lemon-shaped	soil, lepidopterous pupa	Smith 1957
		2.3–2.5(–3) × 1.5–1.8		
*S. aurantia*	cylindrical to ellipsoidal	fusiform	lepidopterous larvae	Mongkolsamrit et al. 2018
	(5–)5.5–8.5(–13) × 2–3	(2–)2.5–3.5(–4) × (1–)1.5(–2)		
*S. inthanonensis*	cylindrical	short fusiform	lepidopterous larvae	Mongkolsamrit et al. 2018
	(4–)6.5–10(–12) × (1–)1.5–2(–3)	(2–)3(–3.5) × 1.5–2		
*S. coleopterorum*	cylindrical to ellipsoidal	fusiform, ellipsoidal or subglobose	snout beetle	this study
	5.4–9.7 × 1.2–1.8	1.7–2.5 × 1.2–1.8		
*S. hymenopterorum*	cylindrical	fusiform to ovoid	bee	this study
	6.5–10.6 × 1.2–2.0	1.9–2.5 × 1.5–2.1		
*S. lepidopterorum*	ellipsoidal	fusiform to subglobose	lepidopterous pupa	this study
	5.2–8.5(–13.1) × 1.1–1.7	2.0–2.5 × 1.2–2.0		

### Samsoniella hymenopterorum

Taxon classificationFungiHypocrealesCordycipitaceae

W.H. Chen, Y.F. Han & Z.Q. Liang sp. nov.

F09AE22C-FF9F-59A3-8F34-59DFC6F23B1D

831736

Fig. 4

#### Diagnosis.

Differs from *Samsoniella inthanonensis* and *S. aurantia* by having smaller, fusiform to ovoid conidia and a host in the family Vespidae.

#### Type.

China, Guizhou Province, Xishui County, at 28°29'56.70"N, 106°24'31.04"E, July 2018, Jiandong Liang, holotype GZAC A1952, ex-type culture GZAC A19522. Sequences from isolated strain A19522 have been deposited in GenBank with accession numbers: ITS = MN128224, *RPB1* = MT642603, *RPB2* = MT642604 and *TEF* = MN101588.

**Figure 4.**
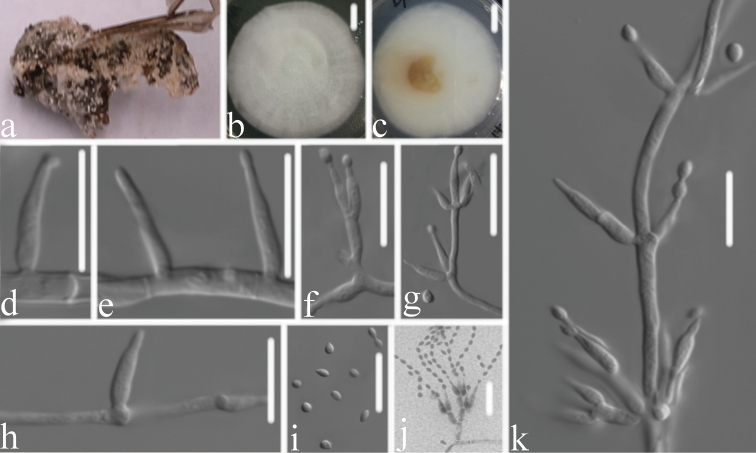
*Samsoniella hymenopterorum***A** infected insect (Hymenoptera) **B, C** top (**B**) and underside (**C**) of a colony cultured on PDA medium at 14 d **D–H, J** phialides and conidia in chains **K** conidiophore and phialides **I** conidia. Scale bars: 10 mm (**B, C**); 10 μm (**D–K**).

#### Description.

Colonies on PDA, 6.2–6.4 cm diam. in 14 d at 25 °C, white, consisting of a basal felt and cottony, floccose hyphal overgrowth, reverse yellowish. Prostrate hyphae smooth, septate, hyaline, 1.1–1.6 μm diam. Erect conidiophores usually arising from aerial hyphae, Isaria-like with phialides in whorls of three to four. Phialides 6.5–10.6 × 1.2–2.0 μm, with a cylindrical basal portion, tapering to a distinct neck. Conidia in chains, hyaline, fusiform to ovoid, 1-celled, 1.9–2.5 × 1.5–2.1 μm. Chlamydospores and synnemata not observed. Size and shape of phialides and conidia similar in culture and on natural substratum. Sexual state not observed.

#### Host.

Bee, family Vespidae.

#### Distribution.

Xishui County, Guizhou Province, China.

#### Etymology.

Referring to its insect host, order Hymenoptera.

#### Remarks.

*Samsoniella hymenopterorum* was identified as belonging to *Samsoniella*, based on the phylogenetic analyses (Fig. 1). Comparing with the typical characteristics of the three species (Table 2), *S. hymenopterorum* has a close relationship with *S. inthanonensis* by a having cylindrical basal in phialide and similar in size. However, it is distinguished from *S. inthanonensis* by having smaller, fusiform to ovoid conidia and a host in the family Vespidae. Based on combined dataset of ITS, *RPB1*, *RPB2* and *TEF* sequences, *S. hymenopterorum* is phylogenetically close to *S. aurantia* and *S. inthanonensis* (Fig. 2). However, *S. hymenopterorum* has smaller fusiform to ovoid conidia and a different host.

### Samsoniella lepidopterorum

Taxon classificationFungiHypocrealesCordycipitaceae

W.H. Chen, Y.F. Han & Z.Q. Liang sp. nov.

09A7269B-030B-5978-86BA-22D4B6378101

831737

Fig. 5

#### Diagnosis.

Differs from *Samsoniella coleopterorum* by having larger, ellipsoidal phialide conidia and a host in the order Lepidoptera.

#### Type.

China, Guizhou Province, Rongjiang County (26°01'56.13"N, 108°24'48.06"E), October 2018, Wanhao Chen, holotype GZAC DL1007 = RJ1807, ex-type culture GZAC DL10071 = RJ18071. Sequences from isolated strain DL10071 have been deposited in GenBank with accession numbers: ITS = MN128076, *RPB1* = MN101592, *RPB2* = MN101593 and *TEF* = MN101594.

**Figure 5.**
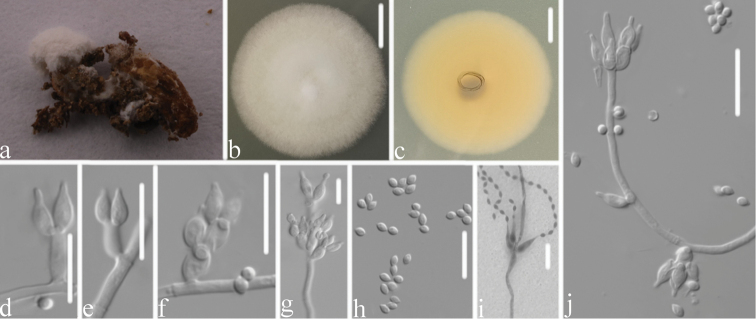
*Samsoniella lepidopterorum***A** infected insect pupa (Lepidoptera) **B, C** top (**B**) and underside (**C**) of a colony cultured on PDA medium at 14 d **D–G, I** phialides and conidia in chains **H** conidia **J** conidiophore and phialides. Scale bars: 10 mm (**B, C**); 10 μm (**D–J**).

#### Description.

Colonies on PDA, 3.7–3.8 cm diam. in 14 d at 25 °C, white, consisting of a basal felt and cottony, floccose hyphal overgrowth, reverse yellowish. Prostrate hyphae smooth, septate, hyaline, 1.1–2.2 μm diam. Erect conidiophores usually arising from aerial hyphae, Isaria-like with phialides in whorls of two to four. Phialides 5.2–8.5 (–13.1) × 1.1–1.7 μm, with an ellipsoidal basal portion, tapering into a distinct neck. Conidia in chains, hyaline, fusiform to subglobose, 1-celled, 2.0–2.5 × 1.2–2.0 μm. Chlamydospores and synnemata not observed. Size and shape of phialides and conidia similar in culture and on natural substratum. Sexual state not observed.

#### Host.

Pupa, order Lepidoptera

#### Distribution.

Rongjiang County, Guizhou Province, China

#### Etymology.

Referring to its insect host, order Lepidoptera

#### Remarks.

*Samsoniella lepidopterorum* was easily identified as belonging to *Samsoniella*, based on the phylogenetic analyses (Fig. 1). Based on the combined dataset of ITS, *RPB1*, *RPB2* and *TEF* sequences (Fig. 2) and the typical characteristics of *Samsoniella* species (Table 2), *S. lepidopterorum* has a close relationship with *S. coleopterorum*. However, *S. lepidopterorum* has larger, ellipsoidal phialide conidia and its pupa host is in the order Lepidoptera.

## Discussion

Phylogenetic analyses, based on the combined datasets of (ITS+*RPB1*+*RPB2*+*TEF*), suggest that the three new species are members of the Cordycipitaceae and belong to the genus *Samsoniella* (Fig. 1). Mongkolsamrit et al. (2018) noted that the typical characteristics of *Samsoniella* were oval to fusiform conidia, bright red-orange stromata of the sexual morphs and synnemata of the asexual morphs. The phialides in this genus range from cylindrical to possessing a swollen basal portion. *S. coleopterorum*, *S. hymenopterorum* and *S. lepidopterorum* all have cylindrical phialides and fusiform conidia. However, the three new species had mononematous conidiophores rather than synnemata. Synnematous entomopathogenic fungi (such as *Gibellula* spp.) can be found on abaxial leaf surfaces of shrubbery, forest floors and shallow soil layers (Hywel-Jones 1996). As air flow under the forest canopy is slow and humid, the dispersal of conidia through airflow diffusion may be difficult. Therefore, these entomopathogenic fungi may employ a particular strategy, such as producing synnemata and sticky conidia, to accommodate various arthropod activities and facilitate conidium spread (Abbott 2002). The three new species were located in the more open portion of the forest and this may favour the dispersal of dry conidia. Thus, we could speculate that the mononematous conidiophores of the three new species may be the result of a convergent evolution to adapt to the ecological environment.

The evolutionary dynamics of fungi and their hosts are usually described either by co-evolution or by host shifts. Shifts often occur to new hosts that are evolutionarily distant, but which occupy a common ecological niche (Vega et al. 2009). Nutrient requirements often determine whether host shifts occur (Vega et al. 2009). Relationships between insects and fungi have been described as biotrophy, necrotrophy and hemibiotrophy, *inter alia*. The common ancestor of Hypocreaceae and Clavicipitaceae corresponds to a departure from plant-based nutrition to one that specialises on animals and fungi (Spatafora et al. 2007). Prediction of the characteristics and evolutionary placement of any given member should be based on the correlation between molecular-phylogenetic genealogy and nutritional preferences (Spatafora et al. 2007; Vega et al. 2009). Species of *Samsoniella* were originally found on lepidopteran larvae or pupae buried in soil or leaf litter (Mongkolsamrit et al. 2018). Mongkolsamrit et al. (2018) also reported that the true range of host affiliations of *Samsoniella* in nature may not be currently represented. Here, we report *Samsoniella* spp. from coleopteran, hymenopteran larvae and lepidopteran pupae. The presence of different hosts indicates that the nutrient requirements of *Samsoniella* spp. can change with the environment (Spatafora et al. 2007).

In the present study, a four loci phylogenetic analysis showed that *S. coleopterorum*, *S. lepidopterorum* and *S. hymenopterorum* clustered in separate subclades from other *Samsoniella* species. They represent new taxa, based on morphological characteristics, nutritional preferences and phylogenetic analyses.

## Supplementary Material

XML Treatment for Samsoniella coleopterorum

XML Treatment for Samsoniella hymenopterorum

XML Treatment for Samsoniella lepidopterorum

## References

[B1] Abbott SP (2002) Insects and other arthropods as agents of vector-dispersal in fungi. http://www.thermapure.com/pdf/AbbottInsectdispersal-2.pdf

[B2] Castlebury LA Rossman AY Sung GH Hyten AS Spatafora JW (2004) Multigene phylogeny reveals new lineage for *Stachybotrys chartarum*, the indoor air fungus. Mycological Research 108: 864–872. 10.1017/S0953756204000607 15449591

[B3] Chen WH Liu C Han YF Liang JD Tian WY Liang ZQ (2019) Three novel insect-associated species of *Simplicillium* (Cordycipitaceae, Hypocreales) from Southwest China. MycoKeys 58: 83–102. 10.3897/mycokeys.58.37176 31592222 PMC6775174

[B4] Darriba D Taboada GL Doallo R Posada D (2012) jModelTest 2: more models, new heuristics and parallel computing. Nature Methods 9(8): 772–772. 10.1038/nmeth.2109 PMC4594756 22847109

[B5] Drummond A Rambaut A (2007) BEAST: Bayesian evolutionary analysis by sampling trees. BMC Evolutionary Biology 7: e214. 10.1186/1471-2148-7-214 PMC2247476 17996036

[B6] Hywel-Jones N (1996) *Akanthomyces* on spiders in Thailand. Mycological Research 9: 1065–1070. 10.1016/S0953-7562(96)80214-0

[B7] Katoh K Standley DM (2013) MAFFT multiple sequence alignment software version 7: improvements in performance and usability. Molecular Biology and Evolution 30(4): 772–780. 10.1093/molbev/mst010 23329690 PMC3603318

[B8] Kepler RM Luangsa-ard JJ Hywel-Jones NL Quandt CA Sung GH Rehner SA Aime MC Henkel TW Sanjuan T Zare R Chen M Li Z Rossman AY Spatafora JW Shrestha B (2017) A phylogenetically-based nomenclature for Cordycipitaceae (Hypocreales). IMA Fungus 8: 335–353. 10.5598/imafungus.2017.08.02.08 29242779 PMC5729716

[B9] Liang JD Han YF Zhang JW Du W Liang ZQ Li ZZ (2011) Optimal culture conditions for keratinase production by a novel thermophilic *Myceliophthora thermophila* strain GZUIFR-H49-1. Journal of Applied Microbiology 110: 871–880. 10.1111/j.1365-2672.2011.04949.x 21241422

[B10] Luangsa-ard JJ Hywel-Jones NL Samson RA (2004) The order level polyphyletic nature of *Paecilomyces* sensu lato as revealed through 18S-generated rRNA phylogeny. Mycologia 96: 773–780. 10.1080/15572536.2005.11832925 21148898

[B11] Mongkolsamrit S Noisripoom W Thanakitpipattana D Wutikhun T Spatafora JW Luangsa-ard J (2018) Disentangling cryptic species with Isaria-like morphs in Cordycipitaceae. Mycologia 110: 230–257. 10.1080/00275514.2018.1446651 29863995

[B12] Ronquist F Teslenko M van der Mark P Ayres DL Darling A Höhna S Larget B Liu L Suchard MA Huelsenbeck JP (2012) MrBayes 3.2: efficient Bayesian phylogenetic inference and model choice across a large model space. Systematic Biology 61: 539–542. 10.1093/sysbio/sys029 22357727 PMC3329765

[B13] Samson RA (1974) *Paecilomyces* and some allied hyphomycetes. Studies in Mycology 6: 1–119.

[B14] Silvestro D Michalak I (2012) raxmlGUI: a graphical front-end for RAxML. Organisms Diversity & Evolution 12(4): 335–337. 10.1007/s13127-011-0056-0

[B15] Smith G (1957) Some new and interesting species of micro-fungi. Transactions of the British Mycological Society 40(4): 481–488. 10.1016/S0007-1536(57)80054-0

[B16] Spatafora JW Sung GH Sung JM Hywel-Jones NL White JF (2007) Phylogenetic evidence for an animal pathogen origin of ergot and the grass endophytes. Molecular Ecology 16: 1701–1711. 10.1111/j.1365-294X.2007.03225.x 17402984

[B17] Swofford DL (2002) PAUP* 4.0b10: phylogenetic analysis using parsimony (*and other methods). Sunderland, MA, Sinauer.

[B18] Tamura K Stecher G Peterson D Filipski A Kumar S (2013) MEGA6: molecular evolutionary genetics analysis version 6.0. Molecular Biology and Evolution 30: 2725–2729. 10.1093/molbev/mst197 24132122 PMC3840312

[B19] Vaidya G Lohman DJ Meier R (2011) SequenceMatrix: concatenation software for the fast assembly of multi-gene datasets with character set and codon information. Cladistics 27(2): 171–180. 10.1111/j.1096-0031.2010.00329.x 34875773

[B20] van den Brink J Samson RA Hagen F Boekhout T de Vries RP (2012) Phylogeny of the industrial relevant, thermophilic genera *Myceliophthora* and *Corynascus*. Fungal Diversity 52: 197–207. 10.1007/s13225-011-0107-z

[B21] Vega FE Goettel MS Blackwell M Chandler D Jackson MA Keller KM Maniania KN Monzón A Ownley BH Pell JK Rangel DEN Roy HE (2009) Fungal entomopathogens: new insights on their ecology. Fungal Ecology 2: 149–159. 10.1016/j.funeco.2009.05.001

[B22] White TJ Bruns T Lee S Taylor J (1990) Amplification and direct sequencing of fungal ribosomal RNA genes for phylogenetics. In: Innis MA Gelfand DH Sninsky JJ White TJ (Eds.) PCR protocols: a guide to methods and applications.Academic Press, New York, 315–322. 10.1016/B978-0-12-372180-8.50042-1

[B23] Zou X Liu AY Liang ZQ Han YF Yang M (2010) *Hirsutella liboensis*, a new entomopathogenic species affecting Cossidae (Lepidoptera) in China. Mycotaxon 111(1): 39–44. 10.5248/111.39

